# Measuring direct non-medical burden among patients with advanced non-small cell lung cancer in China: is there a difference in health status?

**DOI:** 10.3389/fpubh.2023.1090623

**Published:** 2023-05-04

**Authors:** Yu Xia, Yingyao Chen, Jia Chen, Yuying Gan, Chunxia Su, Haibo Zhang, Enwu Long, Fei Yan, Yi Yang

**Affiliations:** ^1^School of Public Health, Fudan University, Shanghai, China; ^2^National Health Commission Key Laboratory of Health Technology Assessment, Fudan University, Shanghai, China; ^3^Department of Medical Oncology, The Affiliated Tumor Hospital of Nantong University, Nantong Tumor Hospital, Nantong, China; ^4^Department of Respiratory Medicine, The Affiliated Hospital of Xuzhou Medical University, Xuzhou, China; ^5^Department of Oncology, Shanghai Pulmonary Hospital, Thoracic Cancer Institute, Tongji University School of Medicine, Shanghai, China; ^6^Department of Organization and Personnel, The First Affiliated Hospital of Nanjing Medical University, Nanjing, China; ^7^Department of Pharmacy, Sichuan Academy of Medical Sciences/Sichuan Provincial People's Hospital, Chengdu, Sichuan, China; ^8^Department of Oncology, Jiangsu Cancer Hospital, Jiangsu Institute of Cancer Research, The Affiliated Cancer Hospital of Nanjing Medical University, Nanjing, China

**Keywords:** direct non-medical cost, health-related quality of life, NSCLC, influencing factors, China

## Abstract

**Objective:**

This study was conducted to estimate the direct non-medical cost of advanced non-small cell lung cancer (NSCLC) patients and explore whether its associated factors vary by health status.

**Methods:**

Data were obtained from 13 centers in five provinces for patients with advanced NSCLC in China. The direct non-medical cost of patients since the patients were diagnosed with NSCLC included the cost of transportation, accommodation, meal, hired caregiving, and nutrition. We measured patients' health status by EQ-5D-5L instrument and divided them into good (≥0.75) and poor (<0.75) groups based on the utility score. A generalized linear model (GLM) was used to assess independent associations between statistically significant factors and non-medical financial burden in health status subgroups.

**Results:**

Data from 607 patients were analyzed. The direct non-medical cost associated with advanced NSCLC since diagnosis was $2,951 per case ($4,060 in the poor health group and $2,505 in the other), with nutrition costing the most. GLM results showed that residence(Urban area vs. Rural area: −1.038, [−2.056, −0.02]), caregivers' occupation type (Farmer vs. Employee: −1.303, [−2.514, −0.093]), hospitalization frequency (0.077, [0.033, 0.12]), average length of hospital stay (0.101, [0.032, 0.17]), and pathological type (Squamous carcinoma vs. Non-squamous carcinoma: −0.852, [−1.607, −0.097]) were independent factors influencing direct non-medical cost in the poor health group. Among participants with good health status, residence (Urban area vs. Rural area: −0.621, [−1.005, −0.236]), marital status (Others vs. Married: 0.762, [0.035, 1.488]), patients' employment status, current caregiving time per day (more than 9 hours per day vs. less than 3 hours per day: 0.471, [0.134, 0.807]), duration of disease (0.015, [0.007, 0.024]), and hospitalization frequency (0.091, [0.068, 0.113]) were statistically associated factors.

**Conclusion:**

The direct non-medical economic burden of advanced NSCLC patients in China is considerable and differs by health status. Strengthening accessibility for more effective therapies and early nutritional intervention to improve prognosis, and further promoting accessible care forms within relevant healthcare insurance coverage may be potentially feasible approaches to alleviate the direct non-medical economic burden for patients and their families.

## Introduction

Lung cancer is the most prevalent new cancer case in China, accounting for about 20.4% (1). Non-small cell lung cancer (NSCLC) constitutes 85–90% of lung cancer diagnoses (2, 3), with the majority of patients in advanced stages (4) and probably having a worse prognosis (5).

Advanced NSCLC is associated with a substantial disease economic burden individual and the healthcare system due to its disease-related impacts, high level of disability inducted, and escalating costs of pharmacological therapy (6). Most studies estimated the direct medical burden among patients with lung cancer (7, 8). Compared with the direct medical cost, the non-medical cost comprises a relatively small proportion of the overall economic burden (9). However, newly emerged immunotherapy and targeted pharmaceuticals have dramatically improved the prognosis and prolonged overall survival for advanced NSCLC patients, potentially leading to an increase in routine anticancer treatment. Therefore, the patients and their families should suffer a considerable share of out-of-pocket direct non-medical expenditures related to frequent therapeutic activities, which are typically not reimbursed by public healthcare insurance schemes (10).

Furthermore, the unfavorable prognosis of cancer and adverse events arising from antineoplastic therapy also contributed to the deterioration in the health-related quality of life (HRQoL) among patients (11, 12). Patients with advanced NSCLC in relatively worse disease conditions usually require more frequent hospital visits and additional support from their caregivers, leading to higher direct non-medical costs such as accommodation and transportation expenses. This implies that poorer HRQoL could be associated with a higher direct non-medical economic burden for advanced NSCLC patients. HRQoL can be regarded as an evaluation index of cancer treatment effectiveness and patient's health status (13), and for patients with advanced NSCLC, enhancing HRQoL and survival are the key targets of treatments (14). Previous studies mainly focused on the measurement of HRQoL in various clinical treatments (15, 16) or exploring its influencing factors among NSCLC patients (17). The correlation between non-medical economic burden and HRQoL, which is the core patient-relevant endpoint generally considered in the benefit assessment of advanced NSCLC treatment (18), also needs to be concentrated.

To the best of our knowledge, there is little research on the direct non-medical burden and its determinants for patients with advanced NSCLC in different health statuses. Therefore, this study aims to examine direct non-medical costs for advanced NSCLC patients with different health statuses and explore the associated factors separately.

## Materials and methods

### Study design

We conducted this hospital-based cross-sectional study in 13 centers as a part of the Demonstration Program on Health Technology Assessment, a nationwide investigation on patients with locally advanced and metastatic NSCLC without sensitizing EGFR and ALK alterations in China. This study protocol was approved by the institutional review board, Public Health School of Fudan University (IRB00002408 and FWA00002399). Written informed consent was obtained from all participants, and all data were anonymized to keep the identity of participants private.

### Study participants

Patients with locally advanced and metastatic NSCLC without sensitizing EGFR and ALK alterations were recruited from November 2020 to June 2021. They were admitted to the Department of Medical Oncology or Respiratory Medicine in sample hospitals within 2 months after the start of this investigation. The enrolled participants were then screened according to inclusion and exclusion criteria. Inclusion criteria were histologically or cytologically confirmed patients with locally advanced or metastatic NSCLC (stage IIIB or IV) without EGFR/ALK mutation, age 18 years or older, and with an ample level of physical and mental health to complete the investigation questionnaire independently or with the assistance of their families. Exclusion criteria were patients who were enrolled in clinical trials, unable to understand the questionnaire, or combined with other serious systemic diseases. Caregivers were also recruited in parallel with the patients. Caregivers had to be family members of the patient, understand the patient's disease condition and medical costs, and be over 18 years of age.

### Survey items and data collection

From the literature review and expert opinions, we independently constructed a structured closed-ended type questionnaire to collect relevant data for the target patients. Data were collected with the questionnaire using a combination of convenience sampling and cluster sampling methods from November 2020 to June 2021 in 13 centers in China, including eight general tertiary hospitals, three regional cancer hospitals, one pulmonary hospital (specialized hospital), and one Chinese traditional medicine hospital from Jiangsu, Shanghai, Fujian, Shandong, and Sichuan Provinces.

All participants were interviewed face-to-face by trained researchers in this study. For patients who had multiple hospitalizations over the survey period, each participant may only take the survey once. Before the formal survey, the questionnaire was pretested, and adjustments and validations were made based on the findings of the pretest. To ensure the quality of the survey, the interviewers explained the questionnaire to each eligible respondent prior to starting the questionnaire, to guarantee that all participants could clearly understand, judge, and answer each question. Furthermore, each questionnaire was carefully checked by interviewers after the survey.

### Dependent variable

#### Estimation of direct non-medical cost

Direct non-medical cost comprises the following aspects: cost of transportation, accommodations, and meals for the patient and their accompanying individuals incurred in the course of NSCLC-related treatment, as well as the cost of the hired caregiving and nutrition during hospitalization and home recuperation of NSCLC patients. The cost of transportation, accommodations, and meals was collected via average cost per visit (including inpatient and outpatient visits), while the cost of nutrition was collected by cost per month. The hired caregiving cost that happened in hospitalization was collected by the average cost per visit, and that incurred in the non-hospitalization period was collected by cost per month. The direct non-medical costs were estimated from the time the patient was diagnosed with advanced NSCLC to the moment of the interview, including the direct non-medical costs related to the diagnostic process.

Hence, the direct non-medical costs of NSCLC patients were computed by the following formula:


$$\begin{array}{l} DNC=DNC_{a}+DNC_{m}+DNC_{t}+DNC_{hc}+DNC_{n} \end{array}$$

where DNC, direct non-medical costs; DNC_a_, costs of accommodation; DNC_m_, costs of meal; DNC_t_, costs of transportation; DNC_hc_, costs of hired caregiving; DNC_n_, costs of nutrition.

The data of direct non-medical costs were collected in Chinese yuan but were converted into US dollars at an exchange rate of 6.45 yuan per dollar (the average annual exchange rate in 2021 from China Foreign Exchange Trade System, https://www.chinamoney.com.cn/chinese/bkccpr/).

### Independent variable

#### Health status

The EQ-5D questionnaire is widely used to measure health-related outcomes and has proven valuable for lung cancer (11). We used the validated Chinese five-level EuroQol-5D scale (EQ-5D-5L) to measure the health status of the NSCLC patients included in the face-to-face interview. The EQ-5D-5L questionnaire included five dimensions (mobility, self-care, usual activities, pain/discomfort, and anxiety/depression), with each dimension having five levels (no problems, slight problems, moderate problems, severe problems, and unable to/extreme problems). We adopted a China-specific scoring algorithm of the EQ-5D-5L in this study, ranging from −0.391 to 1.000 (19, 20).

The EQ-5D utility score was dichotomized into good health status (score ≥ 0.75) and poor health status (score <0.75) based on the literature review (21) and expert opinions. On the one hand, the results of the HRQoL for the included patients showed that the average EQ-5D-5L value in disease progression patients was 0.764. In essence, it is considered that the HRQoL of patients with disease progression or treatment intolerance can be defined as low quality of life (18); On the other hand, referring to Shen et al. (21), the EQ-5D value of NSCLC patients with second-line treatment is 0.768 in China, which generally have a worsening disease or treatment intolerance and poor health status. Therefore, 0.75 was used as the cutoff EQ-5D value for different health status groups.

#### Socio-demographic characteristics

Socio-demographic characteristics included patients' gender, age, residence, marital status, education attainment, occupation type, smoking status, health insurance plan, household income, employment status, and caregiver's occupation type. In the household income variable, we used CNY45,000 ($6,975, referring to the 2019 Chinese urban per capita disposable income) as the baseline option, and the numbers in subsequent options increased in multiples (≤ CNY45,000, CNY45,000-CNY90,000, CNY90,000-CNY180,000, and ≥CNY180,000).

#### Disease- and treatment-related characteristics

The disease- and treatment-related characteristics included hospital type, pathological type, tumor clinical stage, progression of cancer, treatment regimen, gene drive for cancer, duration of the disease since diagnosis, hospitalization frequency, and average length of hospital stay.

### Statistical analysis

Data were presented as frequency (*n*, %) for categorical variables and mean ± SD for continuous variables. We performed a Wilcoxon rank-sum test to compare the direct non-medical cost among different health status participants. The generalized linear model (GLM) with a gamma distribution and log link function was used to explore the predictors of direct non-medical cost for advanced NSCLC. Three models were adopted: Model 1 was applied to compare the direct non-medical cost among good and poor health status participants when controlled for other variables; Models 2 and 3 were used to identify the explanatory factors of non-medical burden in the good and poor health status subgroups, respectively. We also estimated coefficients for the risk factors of five components of direct non-medical cost among two health status subgroups using GLM. An alpha level of 0.05 (two sides) was considered to be statistically significant. And all statistical analyses were performed with R 4.2.1 software.

## Results

### Characteristics of participants

A total of 607 patients were extracted from 13 centers, the mean age was 63.5 years. Most were male individuals (78.6%), over 60 years (63.6%), married (96.0%), and from rural areas (56.3%). Approximately 29% had high school and above education level. Over 30% of the participants and caregivers were employees or farmers. The majority had the lowest annual household income (41.5%), were not working currently (89.3%), had Urban and Rural Residents Basic Medical Insurance (URRBMI, 62.6%), and had over 9 hours caregiving time per day (35.6%).

Regarding the disease-related characteristics, 64.3% of patients were diagnosed with non-squamous carcinoma and 67.1% were metastatic NSCLC patients. In total, 357 were without progression of the disease, 208 received immunotherapy-related regimens (including immunotherapy monotherapy, immunotherapy plus targeted therapy, immunotherapy plus chemotherapy, and immunotherapy plus radiotherapy), and only 51 patients had gene drive. The mean course of NSCLC since diagnosis was 15.44 months, hospitalization frequency was 6.47, and average length of hospital stay was 8.79 days. In total, 433 patients were in the good health status group (EQ-5D-5L utility value ≥ 0.75), whereas 174 reported poor health status. The patient characteristics for the study population are listed in Table 1.

**Table 1:** Socio-demographic and disease-related characteristics.

**Variables**		**Total**	**Poor health status**	**Good health status**
		**(*****n*** = **607)**	**(*****n*** = **174)**	**(*****n*** = **433)**
Total direct non-medical cost^a^ ($, per capita)		2,951	4,060	2,505
Gender	Female	130 (21.4)	36 (20.7)	94 (21.7)
	Male	477 (78.6)	138 (79.3)	339 (78.3)
Age (years)	≤ 60	221(36.4)	57 (32.8)	164 (37.9)
	>60	386 (63.6)	117 (67.2)	269 (62.1)
Residence	Rural area	342(56.3)	105 (60.3)	237(54.7)
	Urban area	265 (43.7)	69 (39.7)	196 (45.3)
Marital status	Married	583 (96.0)	165 (94.8)	418 (96.5)
	Others	24 (4.0)	9 (5.2)	15 (3.5)
Educational attainment	Primary school or lower	218 (35.9)	74 (42.5)	144 (33.3)
	Secondary school	212 (34.9)	59 (33.9)	153 (35.3)
	High school or technical secondary school	131 (21.6)	31 (17.8)	100 (23.1)
	University degree or above	46 (7.6)	10 (5.7)	36 (8.3)
Occupation type (patients)	Employees	76 (12.5)	22 (12.6)	54 (12.5)
	Farmer	109 (18.0)	37 (21.3)	72 (16.6)
	Retiree	190 (31.3)	47 (27.0)	143 (33.0)
	Others^b^	232 (38.2)	68 (39.1)	164 (37.9)
Smoking (years)	Never	232 (38.2)	55 (31.6)	177 (40.9)
	<10	44 (7.2)	11 (6.3)	33 (7.6)
	≥10	331 (54.5)	108 (62.1)	223 (51.5)
Household income ($, per year)	<6,975	252 (41.5)	84 (48.3)	168 (38.8)
	6,975-	196 (32.3)	44 (25.3)	152 (35.1)
	13,950-	113 (18.6)	30 (17.2)	83 (19.2)
	≥27,900	46 (7.6)	16 (9.2)	30 (6.9)
Employment status	Working	13 (2.1)	5 (2.9)	8 (1.8)
	Not working^c^	542 (89.3)	157 (90.2)	385 (88.9)
	Working with occasional sick leave	52 (8.6)	12 (6.9)	40 (9.2)
Insurance^d^	No insurance	6 (1.0)	1 (0.6)	5 (1.2)
	Free medical service	14 (2.3)	3 (1.7)	11 (2.5)
	UEBMI	191 (31.5)	50 (28.7)	141 (32.6)
	URRBMI	380 (62.6)	115 (66.1)	265 (61.2)
	Basic medical insurance + Commercial insurance	16 (2.6)	5 (2.9)	11 (2.5)
Current caregiving time (h/day)	<3	193 (31.8)	25 (14.4)	168 (38.8)
	3–6	142 (23.4)	47 (27.0)	95 (21.9)
	6–9	56 (9.2)	25 (14.4)	31 (7.2)
	>9	216 (35.6)	77 (44.3)	139 (32.1)
Occupation type (caregivers)	Employee	112 (18.5)	30 (17.2)	82 (18.9)
	Farmer	95 (15.7)	38 (21.8)	57 (13.2)
	Retiree	106 (17.5)	29 (16.7)	77 (17.8)
	Others^b^	294 (48.4)	77 (44.3)	217 (50.1)
Hospital type	Specialized hospital^e^	221 (36.4)	74 (42.5)	147 (33.9)
	Traditional Chinese medicine hospital	23 (3.8)	11 (6.3)	12 (2.8)
	General hospital	363 (59.8)	89 (51.1)	274 (63.3)
Duration of disease since diagnosis (months)		15.44 ± 19.89	14.77 ± 16.03	15.71 ± 21.26
Progression	No	357 (58.8)	86 (49.4)	271 (62.6)
	Yes	250 (41.2)	88 (50.6)	162 (37.4)
Hospitalization frequency		6.47 ± 4.37	7.67 ± 5.64	5.99 ± 3.64
Average length of hospital stay (days)		8.79 ± 9.49	8.57 ± 9.35	8.88 ± 9.55
Treatment regimen	Immunotherapy-related therapies^f^	208 (34.3)	56 (32.2)	152 (35.1)
	Others^g^	399 (65.7)	118 (67.8)	281 (64.9)
Clinical stage	Locally advanced (IIIB~IIIC)	200 (32.9)	50 (28.7)	150 (34.6)
	Metastatic (IV)	407 (67.1)	124 (71.3)	283 (65.4)
Gene drive	No	556 (91.6)	162 (93.1)	394 (91.0)
	Yes^h^	51 (8.4)	12 (6.9)	39 (9.0)
Pathological type	Non-squamous carcinoma	390 (64.3)	121 (69.5)	269 (62.1)
	Squamous carcinoma	217 (35.7)	53 (30.5)	164 (37.9)

a The Wilcoxon rank-sum test was used to compare the direct non-medical cost among the two EQ-5D-5L utility value groups.

b Others: included freelancers, the jobless, and others.

c The not working situation includes patients who were retired and those who were employed but completely unable to work due to their illness.

d UEBMI, Urban Employee Basic Medical Insurance; URRBMI, Urban and Rural Residents Basic Medical Insurance; Basic medical insurance, Urban Employee Basic Medical Insurance or Urban and Rural Residents Basic Medical Insurance.

e Specialized hospital included three regional cancer centers and one pulmonary hospital.

f Immunotherapy-related therapies: immunotherapy monotherapy, immunotherapy plus targeted therapy, immunotherapy plus chemotherapy, and immunotherapy plus radiotherapy.

g Others: included chemotherapy, radiotherapy, combination of radiotherapy and chemotherapy, and other treatments.

h Positive driver genes include HER2, KRAS, BRAF, BRCA1/2, ATM, TP53, RET, and TET1.

### The direct non-medical cost of advanced NSCLC patients

**Figure 1:**
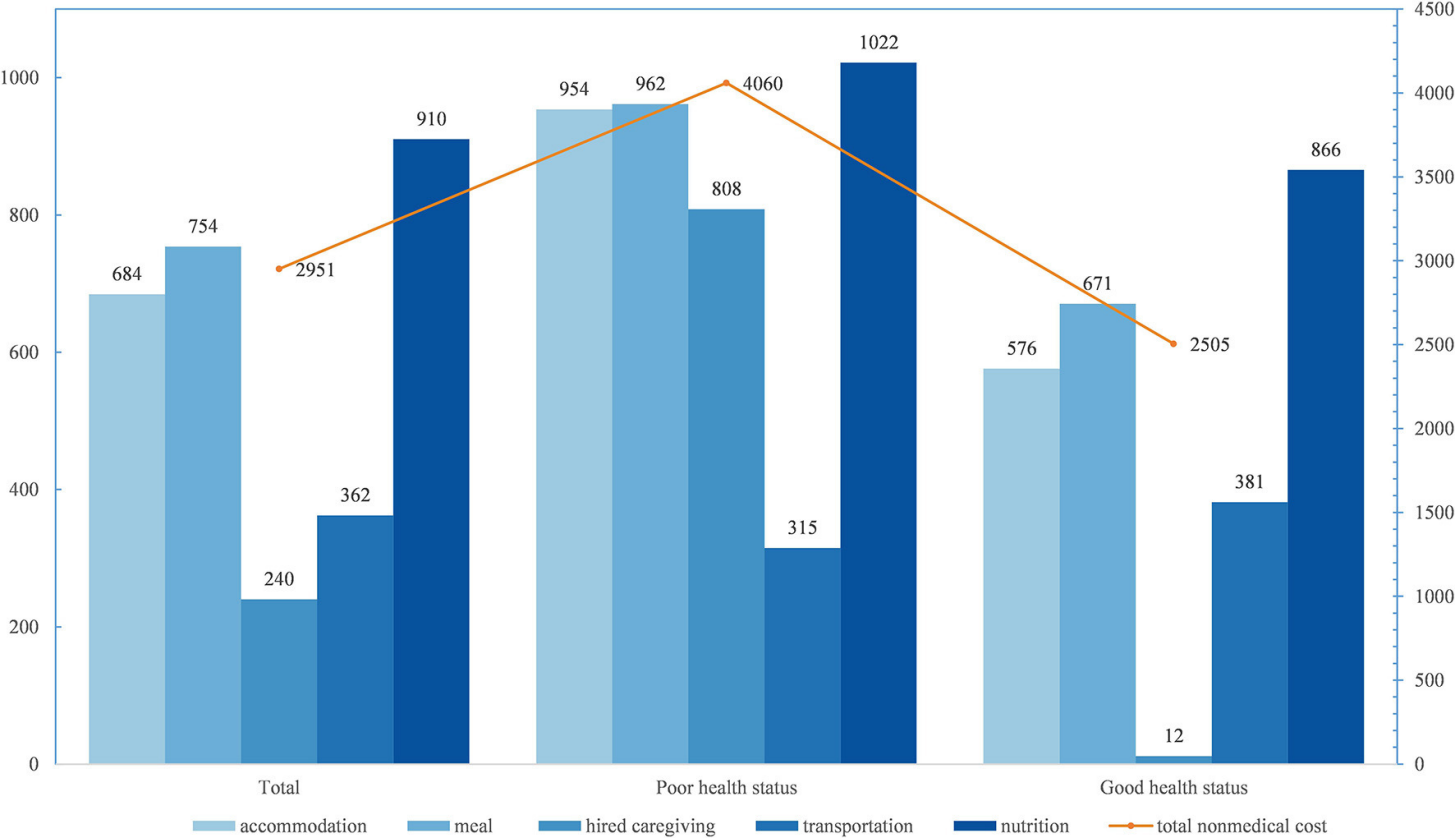
Direct non-medical cost for advanced NSCLC since diagnosis per capita, by health status, USD.

The direct non-medical cost associated with advanced NSCLC since the patient was diagnosed was $2,951 per case (see Figure 1). Among the five components, the cost of accommodation, meals, and nutrition accounted for over 75%, with the highest spending on nutrition (31%). Additionally, the direct non-medical cost per capita in health status subgroups was significantly different (*Z* = 2.356, *P* < 0.05), with $4,060 in poor health status patients and $2,505 in good health status individuals. Compared with poor health status ($808, 19.9% in $4,060), patients with good health status had less caregiving cost.

### Influencing factors of the direct non-medical cost associated with different health statuses

Our results for the association between different health statuses and direct non-medical burdens were presented in Model 1 (Table 2). Even after adjusting for all the confounding factors, patients in good health status were still more likely to have lower direct non-medical costs (B = −0.485, 95% CI: -0.802−0.169).

**Table 2:** Factors affecting direct non-medical costs on patients with advanced NSCLC.

**Variables**	**Model 1** ^a^		**Model 2**		**Model 3**	
	**Beta**	**95% CI**	**Beta**	**95% CI**	**Beta**	**95% CI**
**EQ-5D value**						
<0.75						
≥0.75	−0.485^**^	(−0.802, −0.169)				
**Gender**						
Female						
Male	−0.130	(−0.545, 0.285)	0.099	(−0.961, 1.16)	−0.145	(−0.561, 0.271)
**Age (years)**						
≤ 60						
>60	0.088	(−0.235, 0.41)	−0.339	(−1.259, 0.581)	0.193	(−0.129, 0.515)
**Residence**						
Rural area						
Urban area	−0.473^**^	(−0.863, −0.082)	−1.038^*^	(−2.056, −0.02)	−0.621^**^	(−1.005, −0.236)
**Marital status**						
Married						
Others	0.401	(−0.275, 1.076)	−0.194	(−1.667, 1.279)	0.762^*^	(0.035, 1.488)
**Educational attainment**						
Primary school or lower						
Secondary school	0.151	(−0.196, 0.499)	−0.059	(−0.849, 0.73)	0.308	(−0.05, 0.666)
High school or technical secondary school	0.237	(−0.189, 0.662)	0.455	(−0.511, 1.421)	0.278	(−0.178, 0.735)
University degree or above	−0.004	(−0.675, 0.668)	−1.464	(−3.308, 0.38)	0.390	(−0.298, 1.078)
**Occupation type (patients)**						
Employee						
Farmer	−0.343	(−0.934, 0.249)	0.161	(−1.195, 1.518)	−0.389	(−1.011, 0.233)
Retiree	0.050	(−0.449, 0.548)	0.349	(−0.993, 1.69)	0.115	(−0.37, 0.599)
Others	−0.207	(−0.721, 0.307)	−0.254	(−1.501, 0.994)	−0.031	(−0.553, 0.491)
**Smoking (years)**						
Never						
<10	0.355	(−0.23, 0.94)	0.676	(−0.929, 2.281)	0.118	(−0.442, 0.678)
≥10	0.204	(−0.163, 0.571)	0.240	(−0.705, 1.185)	0.115	(−0.262, 0.492)
**Household income ($, per year)**						
<6,975						
6,975-	0.013	(−0.319, 0.345)	0.165	(−0.672, 1.003)	−0.090	(−0.421, 0.241)
13,950-	0.123	(−0.29, 0.535)	0.454	(−0.669, 1.577)	0.130	(−0.276, 0.537)
≥27,900	0.388	(−0.222, 0.998)	1.169	(−0.251, 2.589)	−0.101	(−0.734, 0.533)
**Employment status**						
Working						
Not working	0.841	(−0.135, 1.817)	−0.014	(−2.183, 2.155)	1.346^*^	(0.281, 2.411)
Working with occasional sick leave	1.398^*^	(0.352, 2.443)	−0.807	(−3.118, 1.504)	2.112^**^	(1.001, 3.223)
**Insurance**						
No insurance						
Free medical service	0.557	(−1.1, 2.214)	0.730	(−3.793, 5.253)	0.456	(−1.098, 2.009)
UEBMI	0.249	(−1.089, 1.587)	−0.389	(−4.186, 3.408)	0.307	(−0.922, 1.536)
URRBMI	0.084	(−1.22, 1.388)	−1.220	(−4.881, 2.441)	0.067	(−1.133, 1.267)
Basic medical insurance + Commercial insurance	−0.269	(−1.784, 1.245)	−0.184	(−4.305, 3.937)	−0.653	(−2.11, 0.803)
**Current caregiving time (h/day)**						
<3						
3–6	0.376	(0.02, 0.731)	0.543	(−0.505, 1.591)	0.341	(−0.006, 0.688)
6–9	0.390	(−0.116, 0.895)	0.212	(−1.009, 1.432)	0.437	(−0.099, 0.972)
>9	0.535^**^	(0.196, 0.875)	0.538	(−0.572, 1.648)	0.471^**^	(0.134, 0.807)
**Occupation type (caregivers)**						
Employee						
Farmer	−0.718^**^	(−1.213, −0.223)	−1.303^*^	(−2.514, −0.093)	−0.394	(−0.925, 0.137)
Retiree	−0.399	(−0.884, 0.086)	−0.435	(−1.765, 0.895)	−0.177	(−0.656, 0.301)
Others	−0.183	(−0.597, 0.231)	−0.149	(−1.334, 1.037)	−0.091	(−0.503, 0.322)
**Hospital type**						
Specialized hospital						
Traditional Chinese medicine hospital	−0.180	(−0.942, 0.581)	−0.299	(−1.911, 1.313)	0.099	(−0.763, 0.961)
General hospital	−0.091	(−0.392, 0.209)	−0.120	(−0.852, 0.612)	−0.030	(−0.332, 0.271)
**Duration of disease since diagnosis (months)**	0.015^**^	(0.006, 0.024)	0.015	(−0.013, 0.043)	0.015^**^	(0.007, 0.024)
**Progression**						
No						
Yes	0.152	(−0.19, 0.494)	0.234	(−0.552, 1.02)	−0.001	(−0.355, 0.353)
**Hospitalization frequency**	0.075^**^	(0.055, 0.095)	0.077^**^	(0.033, 0.12)	0.091^**^	(0.068, 0.113)
**Average length of hospital stay (days)**	0.045^**^	(0.013, 0.076)	0.101^**^	(0.032, 0.17)	0.038	(−0.003, 0.079)
**Treatment regimen**						
Immunotherapy-related therapies						
Others	0.003	(−0.285, 0.29)	0.053	(−0.639, 0.745)	0.068	(−0.22, 0.357)
**Clinical stage**						
Locally advanced (IIIB–IIIC)						
Metastatic (IV)						
**Gene drive**	0.039	(−0.268, 0.346)	−0.107	(−0.84, 0.626)	0.168	(−0.143, 0.48)
No						
Yes	0.003	(−0.501, 0.507)	−0.430	(−1.671, 0.811)	0.146	(−0.372, 0.664)
**Pathological type**						
Non-squamous carcinoma						
Squamous carcinoma	−0.392	(−0.702, −0.081)	−0.852^*^	(−1.607, −0.097)	−0.087	(−0.401, 0.226)

a Model 1 was applied to compare the direct non-medical cost among good and poor health status participants when controlled for other variables; Models 2 and 3 were used to identify the explanatory factors of non-medical burden in the good and poor health status subgroups, respectively.

* P <0.05.

** P <0.01.

It is observed that residence, caregivers' occupation type, hospitalization frequency, average length of hospital stay, and pathological type were independent possible factors influencing the direct non-medical cost in the poor health status group (Model 2). Patients in urban areas had lower direct non-medical burdens (B = −1.038, 95% CI:−2.056-−0.02). Among caregivers' occupation type, farmers were more likely to have lower non-medical costs than employees (B = −1.303, 95% CI:−2.514-−0.093). Patients with higher hospitalization frequency (B = 0.077, 95% CI: 0.033–0.12) and longer length of hospital stay (B = 0.101, 95% CI: 0.032–0.17) were linked to heavier non-medical economic burdens. Compared with non-squamous carcinoma, patients with squamous carcinoma were associated with lower direct non-medical costs (B = −0.852, 95% CI: -1.607-−0.097).

Among participants in good health status, those in urban areas (B = −0.621, 95% CI:−1.005-−0.236) were associated with lower direct non-medical costs. Patients with other marital status (B = 0.762, 95% CI: 0.035–1.488), who were not working or working with occasional sick leave status (*P* < 0.05), had over 9 caregiving hours per day (B = 0.471, 95% CI: 0.134–0.807), longer duration of the disease since diagnosis (B = 0.015, 95% CI: 0.007–0.024), and hospitalization frequency (B = 0.091, 95% CI: 0.068–0.113) were statistically associated with the heavier non-medical financial burden (Model 3).

### Analysis of the composition of non-medical costs by different health statuses

We also explored the influencing factors of five components of non-medical burden for advanced NSCLC patients with good and poor health status, respectively. Patients receiving longer hours of care per day (*P* < 0.05) in good health status were more likely to have higher costs for accommodation, meals, and transportation. The hospitalization frequency (*P* < 0.05) was significantly associated with the cost of accommodation, meals, transportation, and nutrition in both groups. The average length of hospital stay (*P* < 0.05) was the important factor potentially influencing the cost of accommodation, meals, and nutrition among participants in both groups.

Moreover, the longer the duration of the disease since diagnosis was (*P* < 0.05), the heavier the direct non-medical financial burden of nutrition for good health status participants. Urban patients with good health status (*P* < 0.05) were associated with a lower cost of accommodation and transportation. And participants with working employment status (*P* < 0.05) in good health status were more likely to have a lower cost of accommodation, meals, and transportation (see Supplementary Table 1).

## Discussion

The direct non-medical costs of advanced NSCLC patients since diagnosis was $2,951 per capita, with $4,060 in the poor health group and $2,505 in the other. We found that factors influencing direct non-medical costs differ by health status.

The direct non-medical cost of advanced NSCLC patients was a relatively heavy economic burden for patients, which cannot be ignored among the total disease-related economic burden as it is the major out-of-pocket expense outside of healthcare insurance coverage in China (10). Given that the mean disease course since diagnosis of included patients was 15.44 months, the direct non-medical cost per capita per year accounted for almost 46% of disposable income in 2020 in China. The estimated non-medical cost of advanced NSCLC in our research is higher than the prior study on rural tumor patients (22) and a multicenter study on breast cancer patients in China (23), and also higher than similar research in Nepal (24). The reason why our result is higher than others is that it may be influenced by the characteristics of the disease and the level of economic development in regions or countries.

The patient's health status could have a potential impact on the utilization of medical services and relevant costs. Healthcare costs calculated directly using the mean of the overall patient population may obscure the actual effect of patients' health status on the treatment-related cost. Therefore, different health statuses of patients should be distinguished for cost estimation. EQ-5D-5L utility score, as a health status proxy indicator in this study, plays an important role in shaping patients' expectations of their prognosis (15). The good health status group has a relatively lower non-medical cost than the poor group. This may be contributed to the fact that patients with good health conditions may potentially have fewer medical visits for antineoplastic treatment and adverse event management, as well as require less hired nursing and nutritional supplementation.

Similar to previous research, the cost of accommodation, meals, and nutrition in this study account for over 70% of the total direct non-medical cost in two health status subgroups (25), with nutrition expenses accounting for the largest. Nutrition costs from the related health products cover not only the hospital visit but also the daily life during the whole disease course. Cancer is universally acknowledged as a malnourished disease (26), and nutrition status has been found to directly affect both the tolerance and effectiveness of advanced NSCLC treatments. Given that, more attention should be paid to nutrition support to improve the therapeutic effectiveness and HRQoL of NSCLC patients (27) and lessen the related financial burden.

Additionally, this study indicates that five factors (residence, occupation type of caregivers, caregiving time per day, hospitalization frequency, and the average length of hospital stay) are related to the direct non-medical cost of advanced NSCLC patients, and the associated factors varied according to health status subgroups. Among these factors, the occupation type of caregivers and the average length of hospital stay are significantly influencing factors of direct non-medical burden in the poor health status group. Our results also demonstrated that, compared with the employee, families with farmer caregivers, who have a comparatively weak payment capacity for their lower average household income (23), are more likely to have a lower direct non-medical cost of illness. The average length of hospital stay could reflect the severity of the disease (28), which is another indicator affecting the direct non-medical expenses of advanced NSCLC patients with poor health status. The longer the average hospital stay, the higher the direct non-medical cost of patients (29), such as cost for accommodation and meals, which also have a statistically significant correlation within our analysis results on the composition of the non-medical cost.

The significant association between caregiving hours per day and direct non-medical economic burden in the good health status group is supported in our study. Longer caregiving hours per day (more than 9 h) provided by informal caregivers (predominantly family members) are associated with a larger non-medical economic burden (30). An interesting finding in this investigation is that caregiving intensity (caregiving hours per day) did not show any statistically significant correlation with non-medical cost in another health status group. Caregiving intensity could just reflect patients' self-care ability and the severity of their illness to some extent. For patients with relatively poor health conditions, their family members, who are usually full-time and long-term caregivers, provide more personal and intensive daily care to maintain patients' quality of life, which means that direct non-medical costs of these individuals may be insensitive to the changes in caregiving intensity. In contrast, patients with good health status tend to have better self-care capacity and likely require less caregiving. Therefore, the more the daily caregiving hours provided by caregivers, the greater the impact on the recipients' direct non-medical economic burden. Our work also reveals that the longer the disease course, the heavier the direct non-medical financial burden of advanced NSCLC in the good health status group. The non-medical cost collected in our research was cumulative, which was incurred since the patients with advanced NSCLC were diagnosed. Hence, the increase in medical visits triggered by the prolonged course of the disease will lead to an increase in direct non-medical expenditure.

Urban patients have lower direct non-medical costs in both groups in our research. The possible reason is that hospitals with high levels are more likely to be located in cities rather than counties due to the lopsided distribution of health resources between urban and rural China (31). The available condition makes it more convenient for urban patients to have tumor treatment. Therefore, they have lower non-medical costs (such as accommodation and transportation costs). Furthermore, the study demonstrates that hospitalization frequency was an essential factor connected to direct non-medical economic burden in both health status subgroups of advanced NSCLC patients, which was partly consistent with previous studies (29, 32, 33). The increase in hospitalization frequency is accompanied by the increase in direct non-medical expenses generated by accommodation, meals, and transportation, thereby the direct economic burden of hospitalization rises proportionally.

Our research showed the patients' HRQoL has a negative impact on direct non-medical costs. It is suggested that (1) improving patients' prognosis and HRQoL via various ways, such as conducting early screening and detection of cancer programs nationwide and encouraging more innovative targeted interventions with better efficacy to improve timely access to cancer care (23), and other social support suggestions, (2) health institutions, communities, and patients' families should closely monitor patients' nutritional and health condition, and encourage early nutritional interventions, to reinforce patients' immunity and treatment effectiveness, and (3) the government should advocate for formal care (e.g., the community or professional caregiving institutions) for individuals with cancer, and admit this service in the coverage of long-term care insurance to address cancer patients' essential nursing needs.

This study has several limitations. First, this is a cross-sectional study, which could only assess the direct non-medical costs that the participants have already incurred, which is from the time of diagnosis up until the point of the interview, and cannot include the subsequent direct non-medical expenses. Thus, the results of our investigation may underestimate the actual direct non-medical burden for advanced NSCLC patients in China. Second, the individuals with advanced NSCLC in our study were recruited from 13 centers using a combination of convenience sampling and cluster sampling methods, which may not completely reflect the overall patients' situation in China. Finally, recall bias through the patient survey is a potential drawback of our study.

## Conclusion

The direct non-medical economic burden of advanced NSCLC is unignorable, which could result in negative impacts for individual families, and are different in the health status subgroups. These factors, including insurance plan, the average length of hospital stay, hospitalization frequency, occupation type, and caregiving time of caregivers, provide insights to allow targeted intervention to improve HRQoL and lessen the economic burden among cancer patients. Positive measures with particular emphasis are urgent to be undertaken for all advanced NSCLC patients, such as implementing effective early screening and diagnosis measures, early nutritional interventions, and strengthening diverse care forms inside relevant healthcare insurance coverage to potentially reduce the cancer-related economic burden.

## Data availability statement

The informed consent for participants stated that the data was used for this study only and the dataset involved the privacy of participants from multiple institutions. Maybe we cannot share participants data at this time. Requests to access the datasets should be directed to yangyi18@fudan.edu.cn.

## Ethics statement

This study protocol was approved by the Institutional Review Board, Public Health School of Fudan University (IRB00002408 and FWA00002399). The patients/participants provided their written informed consent to participate in this study.

## Author contributions

YX data analysis and wrote the first draft of the manuscript. YY conceived the perspective, material preparation, data collection, participated in design and coordination, and helped to draft the manuscript. CS, JC, EL, HZ, YG, and FY participated in data collection. YC conceived the perspective and contributed to the writing. All authors contributed to the study's conception, design, commented on previous versions of the manuscript, and approved the final manuscript.
