# Supplementary material for: Measuring direct non-medical burden among patients with advanced non-small cell lung cancer in China: is there a difference in health status?
Source: Front Public Health. 2023 May 4;11:1090623. doi: 10.3389/fpubh.2023.1090623 (PMC10192575; doi:10.3389/fpubh.2023.1090623)
Supplement: Supplementary file 1 [file Table_1.pdf]

Supplementary table Factors affecting the composition of direct nonmedical cost on patients with advanced NSCLC

**Variables**	**cost of accommodation**		**cost of meal**		**cost of hired caregiving**		**cost of transportation**		**cost of nutrition**	
	**Poor-**	**Good-**	**Poor-**	**Good-**	**Poor-**	**Good-**	**Poor-**	**Good-**	**Poor-**	**Good-**
Gender										
Female										
Male	1.684	-0.248	-0.126	-0.019	-0.503	-0.262	-0.210	-0.085	1.511*	-2.486**
Age (years)										
≤60										
>60	-0.944	0.094	0.109	0.065	1.518	0.026	-0.497	0.162	-1.541*	1.439**
Residence										
Rural area										
Urban area	0.591	-0.885**	-0.400	-0.275	-1.594	0.439	-0.905	-0.827**	0.112	-2.894**
Marital status										
Married										
Others	-0.097	-0.057	-0.482	-0.112	3.274	6.143**	-0.639	0.460	1.531	2.036*
Educational attainment										
Primary school or lower										
Secondary school	0.454	0.320	0.041	0.033	-0.551	-0.016	-0.200	0.346	0.236	1.361
High school or technical secondary school	1.260	0.590	0.413	-0.043	0.164	-0.664	0.260	0.170	-1.544	1.290*
University degree or above	-7.005**	-0.059	-1.214	-0.198	1.737	-0.223	-2.425**	0.457	-3.447	2.204
Occupation type (patients)										
Employee										
Farmer	3.899**	-0.477	-0.141	-0.413*	3.463	0.854	0.293	-0.204	-1.180	-2.297
Retiree	1.704	-0.341	-0.122	-0.254	3.680*	-0.323	0.587	0.253	-1.372	0.629
Others	3.199**	-0.110	-0.168	-0.148	0.293	-0.259	0.331	-0.011	0.676	-0.977

Smoking (years)										
Never										
<10	-1.327	0.225	0.803	0.086	1.301	-0.801	1.285*	0.119	-2.191	1.392
≥10	0.371	0.041	0.324	0.071	1.310	0.024	0.257	0.091	-2.716**	0.699
Household income ($, per year)										
<6,975										
6,975-	-0.735	-0.146	-0.034	0.142	0.126	0.945*	-0.098	0.033	0.643	-1.398
13,950-	0.936	-0.231	0.996**	0.279*	-2.451	0.277	0.717	0.057	-0.346	-0.846
≥27,900	-0.041	0.177	0.896*	0.109	4.953**	0.181	1.325	-0.156	3.450**	-1.689
Employment status										
Working										
Not working	3.116*	7.249**	0.231	1.216**	0.047	0.282	-0.036	1.387*	-2.660	0.107
Working with occasional sick leave	0.397	7.691**	0.104	1.285**	-1.312	0.523	0.082	2.654**	-6.686**	2.738
Insurance										
No insurance										
Free medical service	10.198**	1.059	-0.515	1.053*	11.166*	7.589**	1.151	-0.515	4.049	1.496
UEBMI	2.094	-0.141	-0.345	0.052	8.509*	1.610	0.771	-0.513	-1.917	2.250
URRBMI	0.236	-0.199	-0.683	-0.003	5.894	1.348	-0.076	-0.897	-2.771	0.617
Basic medical insurance + Commercial insurance	-2.068	-2.590	-0.235	-0.082	3.105	1.924	0.673	-2.080*	-2.143	-0.326
Current caregiving time(h/day)										
<3										
3~6	-0.870	0.400	0.066	0.151	3.072*	1.077*	0.005	0.669**	0.551	0.542
6~9	0.072	0.372	0.261	0.370*	-0.495	1.202	0.484	0.902	0.749	-0.887
＞9	-0.458	0.479*	0.374	0.299**	1.096	1.027	0.572	0.838**	2.119**	1.531**
Occupation type (caregivers)										
Employee										

Farmer	-1.008	-0.393	-0.426	-0.295	-4.402**	-0.927	-0.575	-0.270	-4.527**	-0.267
Retiree	-0.762	-0.447	0.142	-0.281	-3.056	-0.696	0.132	-0.367	-2.277*	0.672
Others	-0.986	-0.528	0.033	-0.207	-0.846	-0.035	0.336	-0.050	-2.894**	0.668
Hospital type										
Specialized hospital										
Traditional Chinese medicine hospital	0.386	0.632	-0.155	0.379	5.201**	-1.075	-0.523	-0.213	-5.516**	3.813
General hospital	-0.235	-0.242	0.110	-0.056	0.447	-1.517**	-0.122	-0.442*	0.645	0.302
Duration of disease since diagnosis (months)	-0.038	0.010	0.004	0.001	0.079	0.000	-0.013	-0.003	0.048	0.059**
Progression										
No										
Yes	0.398	-0.394	-0.319	0.308**	-1.660	-0.580	-0.033	0.346	2.235**	-2.298**
Hospitalization frequency	0.094**	0.097**	0.099**	0.088**	-0.028	0.009	0.148**	0.108**	0.117**	0.113**
Average length of hospital stay (days)	0.147**	0.067*	0.130**	0.131**	0.049	0.017	0.032	-0.084**	0.095*	-0.297**
Treatment regimen										
Immunotherapy-related therapies										
Others	-0.452	0.101	0.061	-0.028	-0.689	-0.573	-0.060	-0.099	0.499	0.545
Clinical stage										
Locally advanced (IIIB~IIIC)										
Metastatic (IV)	-0.475	0.072	-0.105	0.059	-2.221*	-0.303	0.126	-0.155	3.070**	0.793
Gene drive										
No										
Yes	-0.963	0.396	0.012	-0.086	-1.541	-0.567	0.247	0.625	-1.130	0.026
Pathological type										
Non-squamous carcinoma										
Squamous carcinoma	-0.656	0.059	-0.344	-0.035	-2.139**	0.111	-0.872**	-0.158	-0.642	0.289

*P<0.05 **P<0.01
